# Diagnostic challenges in mitochondrial encephalomyopathy with m.10158T>C mutation: a case report and literature review

**DOI:** 10.3389/fnint.2025.1709380

**Published:** 2026-01-16

**Authors:** Zhuqing Luan, Zhigang Liang

**Affiliations:** Department of Neurology, Yantai Yuhuangding Hospital Affiliated to Qingdao University, Yantai, Shandong, China

**Keywords:** case report, mitochondrial DNA, point mutation, mitochondrial encephalomyopathy, stroke, stroke-like episodes

## Abstract

Mitochondrial encephalomyopathy is a complex disorder with heterogeneous clinical manifestations that often complicate its clinical diagnosis. We report the first documented case of a 52-year-old woman harboring a novel and rare genotypic combination: the m.10158T>C point mutation together with a 12.8-kb large-scale mtDNA deletion. After a protracted diagnostic course involving multiple prior misdiagnoses, the definitive diagnosis was ultimately established through integrated genetic, histopathological, and neuroimaging evaluation. This case underscores both the diagnostic challenges in mitochondrial disorders and the critical need for systematic differentiation from common neurological mimics such as encephalitis and stroke.

## 1 Introduction

Mitochondria serve as the primary hub for cellular energy metabolism in eukaryotic organisms, with oxidative phosphorylation (OXPHOS) generating over 90% of the body’s adenosine triphosphate (ATP) demand (Lightowlers et al., 2015). Despite encoding only 37 genes (Gonçalves, 2019), mitochondrial DNA (mtDNA) remains a crucial locus for inherited disorders due to its structural vulnerabilities (Falkenberg et al., 2024; Taylor and Turnbull, 2005; Sharma and Sampath, 2019). Pathogenic mtDNA variants occur in approximately 1 in 5,000 individuals (Orsucci et al., 2021). It leads to respiratory chain dysfunction and impaired energy metabolism (Gorman et al., 2016), predominantly affecting the nervous and muscular systems. Clinically, mitochondrial encephalomyopathy, lactic acidosis, and stroke-like episodes (MELAS) syndrome represents the most frequent phenotype (Tetsuka et al., 2021; El-Hattab et al., 2015; Na and Lee, 2024). This report details the diagnostic odyssey of a middle-aged woman with mitochondrial encephalomyopathy. Over a four-year clinical course, she was initially misdiagnosed with viral encephalitis and later with cerebral infarction, definitive diagnosis was ultimately achieved through genetic testing, which identified the rare m.10158T>C mutation and a large-scale mtDNA deletion. We outline this protracted journey, discuss the challenges in differentiating MELAS from common neurological disorders, and emphasize the importance of early suspicion and comprehensive evaluation.

## 2 Patient information

**Figure 1:**
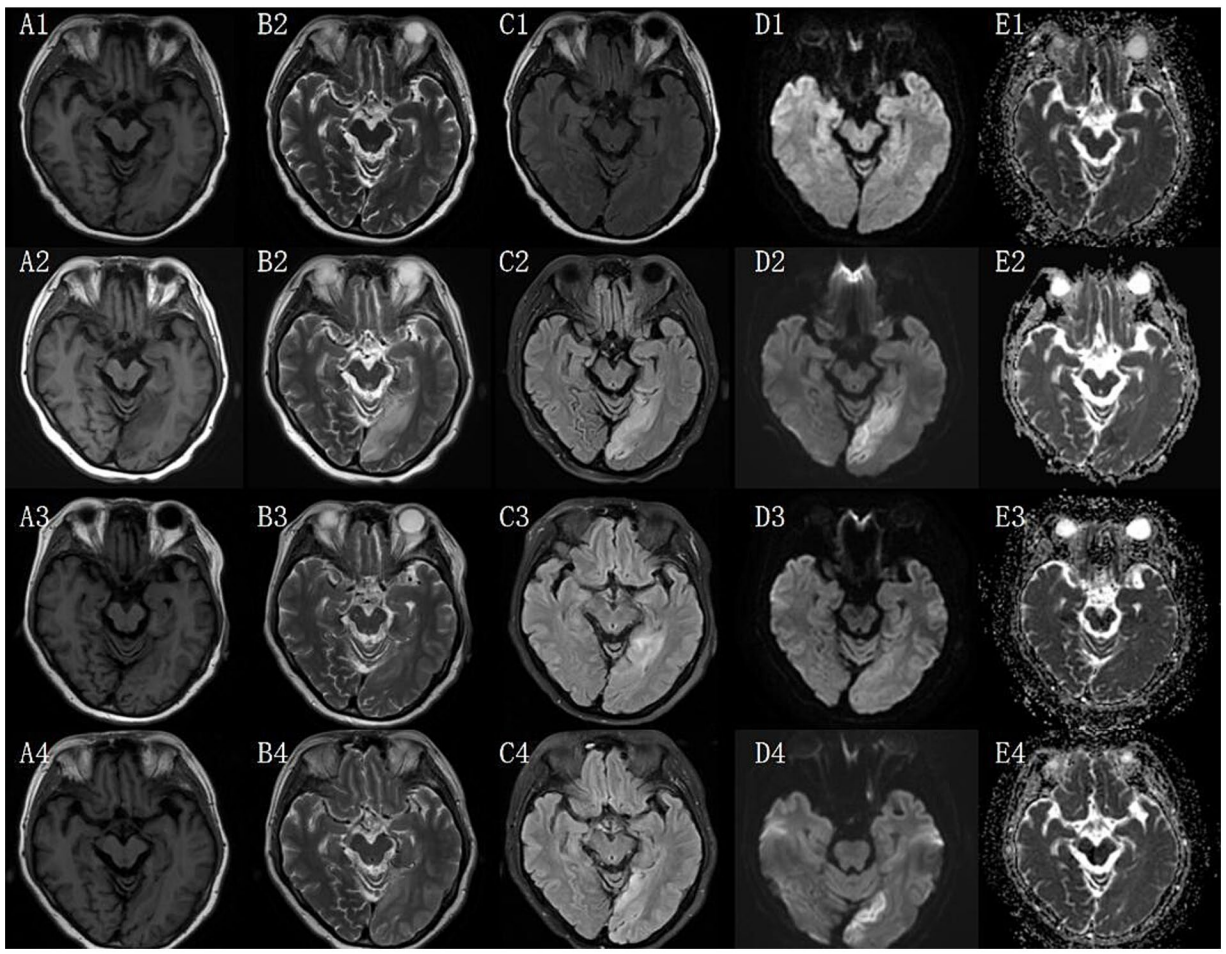
Cranial brain scan + DWI + contrast enhancement (3 T). T1, T1-weighted imaging; T2, T2-weighted imaging; FLAIR, fluid-attenuated inversion recovery; DWI, diffusion-weighted imaging; (A1–E1) 2021/11/21 cranial MRI; (A2–E2) 2024/07/24 cranial MRI; (A3–E3) 2024/08/08 cranial MRI; (A4–E4) 2025/01/10 cranial MRI.

A 52-year-old woman was initially admitted on November 2, 2021, with symptoms of unsteady gait and limb twitching. She was previously healthy with no history of chronic illness, surgery, or medication use prior to this episode, and reported no known allergies. The patient did not smoke or consume alcohol. She had never experienced similar neurological events before. The family history was noncontributory, with no genetic disorders or unusual developmental features reported among immediate family members. Neurological examination revealed altered consciousness, unresponsiveness to questioning, neck stiffness, and sluggish pupillary light reflexes bilaterally. Based on imaging evidence of bilateral frontoparietal white matter demyelination (Figure 1, A1–E1) and elevated CSF protein (599.9 mg/L), a preliminary diagnosis of intracranial infection was established. She was diagnosed with viral encephalitis and discharged on November 17, 2021, after stabilization with antiviral and antiepileptic therapy.

Approximately two and a half years after the initial diagnosis and treatment for presumed viral encephalitis, the patient was readmitted on July 24, 2024, due to recurrent convulsions. Neurological examination revealed impaired consciousness, sluggish pupillary light reflexes, nystagmus with horizontal and vertical gaze, dysarthria, grade 4 muscle strength in the right upper limb, grade 3 muscle strength in the right lower limb, normal strength in the other limbs, and bilateral pathologic signs (+). CSF protein levels were elevated (547.0 mg/L). Cranial MRI (Figure 1, A2–E2) showed patchy long T1 and long T2 signals in the left medial temporal and occipital lobes, with high signals in diffusion-weighted imaging (DWI) and fluid-attenuated inversion recovery (FLAIR). Despite the absence of vascular occlusion on MRA, the imaging pattern led to a working diagnosis of “cerebral infarction,” and she was discharged on August 6, 2024, following a regimen of antiplatelet therapy and lipid regulation.

While the patient’s two prior treatments at outside institutions provided transient symptomatic relief, without elucidating the cause, thereby setting the stage for inevitable symptom recurrence.

## 3 Clinical findings

It was against this backdrop, the patient was admitted to our department on August 8, 2024—her third overall hospitalization—for right limb immobility of 1 day’s duration. Neurological examination revealed rightward tongue deviation, grade 4 muscle strength in the right lower limb, instability during finger-nose and heel–knee-tibia testing, and a positive pathological sign on the left side. Following the unremarkable external MRA, venous evaluation with magnetic resonance venography (MRV) likewise showed no vascular abnormalities. Cranial MRI (Figure 1, A3–E3) demonstrated patchy, mildly prolonged T1 and T2 signal shadows in the left parieto-occipito-temporal lobe, which appeared hyperintense on DWI. A corresponding high-signal focus in the left thalamus showed low signal on apparent diffusion coefficient (ADC) mapping.

**Figure 2:**
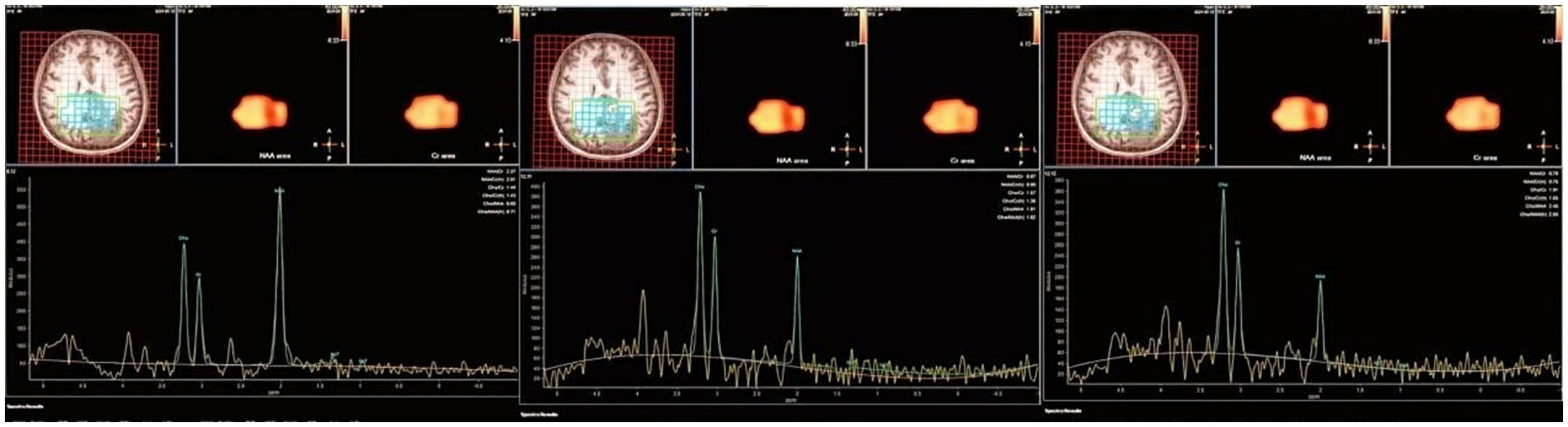
Magnetic resonance spectroscopy (MRS). MRS: Magnetic resonance spectroscopy; NAA/(Cho + Cr): N-acetylaspartate/(choline+creatine). Spectrum analysis demonstrated a significantly reduced N-acetylaspartate (NAA) peak and an elevated lactate (Lac) peak, consistent with mitochondrial dysfunction. The creatine (Cr) peak was mildly decreased. The choline (Cho) peak, while also decreased in absolute terms, was relatively preserved compared to the marked reduction in NAA and the mild reduction in Cr. This is directly quantified by the elevated Cho/NAA and Cho/Cr ratios, with a concurrent decrease in NAA/Cr.

Despite initial presentations that mimicked common cerebrovascular or infectious etiologies, the recurrence of stroke-like episodes alongside elevated CSF protein prompted suspicion of an underlying metabolic disorder. This suspicion was critically supported by an exercise lactate test, which showed a striking elevation (10.6 mmol/L) with post-exercise normalization. The investigation deepened with electroencephalography (EEG) revealing abnormal discharges, and magnetic resonance spectroscopy (MRS) (Figure 2) unveiled a distinct metabolic profile in the lesions, demonstrating: an elevated lactate (Lac) peak; significantly reduced N-acetylaspartate (NAA) and creatine (Cr) peaks; and relative preservation of the choline (Cho) peak. Consistent with these changes, the metabolite ratios were altered, showing elevated Cho/NAA and Cho/Cr with a reduced NAA/Cr. Together with the clinical presentation, these investigative findings firmly supported the diagnosis of a metabolic encephalopathy.

### 3.1 Diagnostic assessment

Based on the constellation of clinical and investigative findings, the case was characterized by: a middle-aged female with recurrent stroke-like episodes, including episodes of impaired consciousness, limb weakness, and convulsions. Neurological findings included altered consciousness, unresponsiveness, and pathologic signs. Cranial MRI revealed patchy high-signal foci in the parieto-occipito-temporal regions without evident vascular abnormalities. EEG abnormalities and MRS spectroscopy indicated reductions in NAA and Cr peaks, alongside elevated lactate, suggestive of mitochondrial dysfunction.

Given the clinical presentation and the history of two prior misdiagnoses, differentiation from viral encephalitis and ischemic stroke was warranted. However, the absence of fever, meningeal signs, or CSF virologic evidence argued against encephalitis. And imaging revealed ischemic lesions, the lack of vascular risk factors and corresponding vascular territory on MRA, coupled with the dynamic, multifocal nature of the lesions and the distinctive MRS profile (elevated lactate), pointed away from typical cerebral infarction. These features collectively favored a metabolic etiology, specifically mitochondrial encephalomyopathy, guiding subsequent genetic testing for definitive diagnosis.

Additionally, autoimmune encephalitis and primary angiitis of the central nervous system (PACNS) were considered important differential diagnoses to exclude. In this case, testing for relevant autoantibodies (e.g., anti-NMDA receptor, anti-LGI1) in serum and cerebrospinal fluid was negative, and there was no history of associated malignancy, thus providing no key evidence to support autoimmune encephalitis. Furthermore, high-resolution magnetic resonance vessel wall imaging revealed no characteristic findings of vasculitis, such as vessel wall thickening or enhancement, and systemic inflammatory markers including erythrocyte sedimentation rate and C-reactive protein were not significantly elevated, which did not support a diagnosis of PACNS.

Together, these findings confirmed the diagnosis of mitochondrial encephalomyopathy and formed the basis for initiating targeted therapeutic intervention.

### 3.2 Therapeutic intervention

These diagnostic findings directly guided the initiation of targeted therapy. Prior to genetic confirmation, a tailored antiseizure and metabolic support regimen was begun based on the converging clinical, metabolic, and radiological evidence of mitochondrial dysfunction. Levetiracetam (750 mg twice daily) served as the foundational broad-spectrum antiseizure agent, chosen for its reliable efficacy and favorable mitochondrial safety profile. To augment seizure control while addressing comorbid neuropathic pain and muscular hypertonia, pregabalin (75 mg twice daily) was incorporated. Lamotrigine was initiated at a low dose (25 mg daily) and carefully up-titrated to further modulate cortical excitability and support long-term seizure stability. And flunarizine (5 mg nightly) was administered prophylactically for migraine-like headaches. To mitigate oxidative stress and support mitochondrial bioenergetics, edaravone (30 mg twice daily, administered intravenously) and coenzyme Q10 (200 mg three times daily) were concurrently administered. This combination avoided mitochondrial-toxic antiseizure medications such as valproate. The patients clinical condition stabilized on this integrated protocol, leading to discharge on August 10, 2024, with a plan for continued oral therapy. Subsequent genetic identification of the mitochondrial chrMT: 10158 T>C mutation validated the treatment approach.

### 3.3 Follow-up and outcomes

The patient was discharged from the hospital with oral levetiracetam antiepileptic treatment, with gradual symptomatic relief. To further characterize the etiology, a underwent mitochondrial long-range PCR (muscle tissue) assay (assay: PCR library construction + high-throughput sequencing) (Table 1): 16,569 loci in the whole mitochondrial genes of the subject, and an abnormality was found in the mitochondrial chrMT: 10158 T>C locus (the nucleotide 10,158 on the mitochondrial genome was mutated from thymine to cytosine, with a mutation ratio of 0.67). And a large deletion variant was found in the mitochondrial genome, with a deletion region of chrMT-3263-16075, a length of 12,812 bp, and containing 33 genes. For comparison, a muscle biopsy obtained from her son showed no significant abnormalities.

**Table 1:** Specific results of testing.

Genetics	Transcription version Excon location	Variant loci (GRCh and 37/hg19)	Genotype^1^, Sequencing depth, Variant allele frequency (VAF)	Allele frequence^2^	Family segregation analysis	ACMG classification^3^	Inheritance^4^
MT-ND3	–	m.10158T>C chrMT-10158	2511/4692 0.65	0	No variant was detected at this locus in his son	Pathogenic	Leigh disease/MELAS

^1^Genotype: Hom: Homozygous variant; Het: Heterozygous variant; Hem: Hemizygous variant; ^2^Population allele frequency: The frequency of this variant in the global population and East Asian population based on sequencing data from the gnomAD database; ^3^ACMG Classification: Pathogenicity assessment follows guidelines from the American College of Medical Genetics and Genomics (ACMG) with the following categories; P (Pathogenic): The variant is classified as pathogenic; LP (Likely Pathogenic): The variant is likely pathogenic; VUS (Variant of Uncertain Significance): The variant has uncertain clinical significance; LB (Likely Benign): The variant is likely benign; B (Benign): The variant is classified as benign; ^4^Inheritance patterns: AR: Autosomal recessive inheritance; AD: Autosomal dominant inheritance; XL: X-linked inheritance; XD: X-linked dominant inheritance; XR: X-linked recessive inheritance.

Following the identification of the mitochondrial chrMT: 10158 T>C mutation, a more targeted differentiation from the Leigh/MELAS overlap syndrome was undertaken. Our patient presented in adulthood with recurrent stroke-like episodes and seizures, a clinical profile aligning with MELAS rather than the infantile-onset developmental regression and brainstem dysfunction characteristic of Leigh syndrome. Crucially, neuroimaging revealed asymmetric, cortically based lesions that migrated over time, without the symmetrical basal ganglia or brainstem involvement pathognomonic of Leigh syndrome. While this specific mutation is associated with a broader phenotypic spectrum, the combined clinical and radiological features in this case nevertheless strongly favor a diagnosis of MELAS. With this definitive molecular diagnosis, her therapeutic management—centered on oral antiepileptic and symptomatic drugs—was therefore maintained as the appropriate course of treatment.

In January 2025, the patient was readmitted for the fourth time due to a recurrence of “right-sided limb immobility and generalized tonic convulsions” and a review of the craniocerebral scanning+DWI + enhancement (3 T) showed (Figure 1, A4–E4): the same region of the left parieto-occipito-temporal lobe was still seen with long T1 and T2 signals, and a Flari high signal shadow. The EEG showed persistent epileptiform discharges over the left parieto-occipito-temporal region. Upon commencement, he severe presenting symptoms of limb rigidity and convulsions were controlled, with no recurrence throughout her stay. The residual clinical picture was limited to occasional headache and intermittent difficulty recognizing family members, consistent with the progression of mitochondrial encephalomyopathy. After achieving stability on the multi-drug regimen, she was discharged on continued oral therapy.

At one-month follow-up, the patient exhibited residual right lower-limb weakness, dysarthria, and mild cognitive impairment. Over the ensuing 6 months, she adhered strictly to the prescribed antiepileptic and neurotrophic regimen, which resulted in freedom from seizure recurrence. Notably, she achieved significant functional recovery and regained full independence in daily living.

### 3.4 Timelines

The diagnostic journey of this 52-year-old woman spanned over 3 years (November 2021 to January 2025), marked by two critical misdiagnoses before definitive resolution (Table 2). Initial presentation led to a misdiagnosis of viral encephalitis (November 2021), highlighting the syndrome’s mimicry of infectious processes. A subsequent admission for seizures was attributed to cerebral infarction (July 2024), underscoring its overlap with vascular disorders. The definitive diagnosis of mitochondrial encephalomyopathy was established in August 2024 following metabolic profiling (elevated lactate on MRS) and later genetically confirmed (m.10158T>C mutation). Despite experiencing a recurrence of seizures and right-sided weakness leading to hospitalization in January 2025, the patient responded well to combined multi-drug therapy, achieving effective symptom control. At follow-up, although she exhibited persistent right lower limb weakness, dysarthria, and mild cognitive impairment, these sequelae did not compromise her functional independence, and she remained fully self-sufficient in activities of daily living.

**Table 2:** Timeline of diagnostic and therapeutic course.

Date	Key event	Clinical significance/Outcome
November 2, 2021	Initial admission: Unsteady gait, limb twitching. Misdiagnosed as viral encephalitis.	Received antiviral and antiepileptic therapy; symptoms stabilized temporarily, but underlying etiology remained unknown.
July 24, 2024	Readmission: Recurrent seizures. Misdiagnosed as cerebral infarction.	Treated with antiplatelet and lipid‑lowering agents; discharged with transient relief, followed by symptom recurrence.
August 8, 2024	Third admission: Comprehensive metabolic workup. MRS shows elevated lactate. Clinical diagnosis of mitochondrial encephalomyopathy.	Empirical regimen started: levetiracetam, pregabalin, lamotrigine, edaravone, coenzyme Q10; condition stabilized, discharged on oral therapy.
2024 (post-discharge)	Genetic testing confirms m.10158T>C mutation and large mtDNA deletion in muscle tissue.	Diagnosis validated; empirical treatment confirmed and maintained as long‑term management.
January 2025	Fourth admission: Recurrence of symptoms. Stabilized on established multi‑drug regimen.	Acute symptoms controlled with continued multi‑drug regimen; discharged on sustained oral therapy.
Follow-up (up to 6 months)	Seizure‑free with residual deficits. Maintains functional independence.	Outcome: Adherence to antiepileptic and neurotrophic regimen resulted in seizure freedom and preserved functional independence in daily living.

## 4 Discussion

This report presents a MELAS case with a distinct genetic architecture: the co-occurrence of the m.10158T>C point mutation and a 12.8-kb large-scale mtDNA deletion, a combination not previously documented. Although isolated large deletions are known to cause MELAS, and the common A3243G mutation has been reported alongside multiple deletions (Aharoni et al., 2010), the specific association of the rare MT-ND3 m.10158T>C variant with a single large deletion has not been described, defining a novel genotypic subtype (Campos et al., 1995). This “dual-hit” genotype suggests a synergistic pathogenic mechanism: the point mutation, present at high heteroplasmy (67% in muscle), likely acts as the primary phenotypic driver, while the coexisting large deletion—even at low abundance—may further deplete the functional mitochondrial genome pool, exacerbating bioenergetic deficit and contributing to the severity of stroke-like episodes.

The diagnostic odyssey in this patient underscores that MELAS is often a diagnosis of exclusion. Key distinctions from common mimics included the absence of infectious or inflammatory markers against viral and autoimmune encephalitis, and the non-territorial, dynamic nature of lesions—coupled with a lack of vascular risk factors or vasculitic changes—which argued against ischemic stroke or primary CNS angiitis. Although the identified m.10158T>C mutation can manifest as a Leigh/MELAS overlap syndrome, the adult onset, cortical-predominant imaging, and clinical phenotype were definitive for classic MELAS. Thus, the conclusive diagnosis rested on integrating negative exclusionary findings with positive metabolic (elevated lactate on MRS) and genetic evidence, highlighting the necessity of a systematic, multimodal diagnostic approach (Finsterer, 2019).

MELAS typically presents in childhood, and clinical symptoms are highly heterogeneous (Hu and Lu, 2023; Zhuo et al., 2023). Stroke-like episodes are the hallmark, with vascular abnormalities, disruption of the blood-cerebrospinal fluid barrier, and nitric oxide deficiency contributing to their pathogenesis. Recurrent episodes lead to progressive decompensation, resulting in complications like respiratory failure, prolonged immobility, and mortality (Yatsuga et al., 2012; Lin et al., 2024). The underlying pathophysiology involves multiple interconnected mechanisms: mitochondrial DNA mutations impair oxidative phosphorylation, leading to lactic acidosis and predisposing to stroke-like episodes (El-Hattab et al., 2015); compensatory anaerobic glycolysis exacerbates metabolic acidosis; abnormal mitochondrial proliferation in cerebral vessel walls compromises vascular function, worsening ischemia (Yang et al., 2018); and energy deficiency reduces nitric oxide synthesis, impairing vasodilation and perfusion (El-Hattab and Jahoor, 2017). Clinical manifestations primarily affect tissues with high energy demands, such as skeletal muscle, the brain, and cardiac muscle (Wang and Le, 2015). In summary, the clinical triad of stroke-like episodes, headache, and seizures constitutes the core phenotype (Wang et al., 2020a).

Reviewing previous literature, the MT-ND3 m.10158T>C mutation is a significant causative locus for mitochondrial encephalomyopathies, impairing Complex I function and leading to energy metabolism disorders (Table 3). Atsuko Kori et al. first reported that patients with this mutation might experience an age-dependent transition from Leigh Syndrome to MELAS, suggesting that the role of Complex I in brain development changes with age (Kori et al., 2019). The heteroplasmy rate varies considerably across tissues. In this case, peripheral blood testing was negative while muscle tissue showed 67% heteroplasmy, indicating that single-tissue testing may yield false-negative results.

**Table 3:** Summary of representative case reports and studies involving m.10158T>C mutation in MT-ND3 gene.

Literature/Year	Patient age	Clinical presentation	Gene mutation (heteroplasmy rate)	Imaging features	Treatment and prognosis
Finsterer and Zarrouk-Mahjoub (2017)	9 years, Male	Leigh-MELAS overlap (headache, WPW syndrome, multi-organ involvement)	Muscle: 95%; Urinary, epithelium: 95%; Oral cells: 80%; Lymphocytes: 70%	Basal ganglia and brainstem lesions, cardiac abnormalities (WPW)	No specific treatment, poor prognosis
Kori et al. (2019)	4 years, Female	Infantile Leigh syndrome (developmental delay), transition to MELAS at age 4 (refractory epilepsy, stroke-like episodes)	Blood: 82% (not detected in parents)	Basal ganglia, brainstem, multifocal white matter and cortical lesions	Levetiracetam, L-arginine; rapid progression, requiring tube feeding
Wang et al. (2020a) and Wang et al. (2020b)	22 years, Female	MELAS (seizures, hemianopia, elevated lactate)	Blood/Hair follicle: m.10158T>C (heteroplasmy rate not reported), parents negative	Bilateral occipital/parietal lesions, DWI hyperintensity (non-vascular distribution)	Coenzyme Q10, arginine therapy; partial symptom relief
Mukai et al. (2017)	41 years, Male	MELAS (stroke-like episodes, epilepsy)	Muscle: 69%, Blood not tested	Normal muscle biopsy, cortical stroke-like lesions	Carbamazepine, levetiracetam; cognitive deterioration

We acknowledge a diagnostic limitation: a comprehensive pathological characterization of the index patient’s muscle biopsy—including specific stains for RRF, COX, and SDH—was not available. Although the presence of “ragged red fibers” (RRFs) is a hallmark, traditional diagnostic approaches that rely solely on such pathological markers remain limited. Genetic testing can confirm the diagnosis even in the absence of RRFs, and overreliance on them may result in missed diagnoses of MELAS with atypical pathology (Wang et al., 2020b; Mukai et al., 2017). Furthermore, MELAS often involves multiple organ systems (Wang and Le, 2015). Finsterer et al. have highlighted the importance of screening for cardiomyopathic lesions and metabolic abnormalities (Finsterer and Zarrouk-Mahjoub, 2017). Restricting the diagnostic focus to neurological symptoms without evaluating systemic indicators like lactic acidosis carries a high risk of misdiagnosis.

Currently, there are no consensus-based curative therapies. Clinical management primarily focuses on controlling epilepsy, managing stroke-like episodes and dystonia, preventing cognitive decline, improving quality of life, and reducing mortality (Ng and McFarland, 2023). In seizure management, mitochondrial-toxic drugs like valproic acid should be avoided, as they may worsen metabolic disturbances (Mukai et al., 2017). Medications with minimal impact on mitochondrial function, such as levetiracetam, should be prioritized, and non-pharmacological interventions like ketogenic diets may be considered (Zweers et al., 2021). Although emerging studies have explored the efficacy of arginine, citrulline, coenzyme Q10, and carnitine (El-Hattab et al., 2015; Barros et al., 2024), recent reports on L-arginine and taurine to prevent stroke-like episodes are promising. L-Arginine, a nitric oxide precursor, can reduce episode frequency and improve symptoms (Koga et al., 2005; Stefanetti et al., 2022). At the molecular level, taurine has been shown to improve respiratory chain dysfunction in MELAS (Ueda et al., 2023), and novel approaches to restore tRNA modifications bring new hope for targeted therapies (Tomoda et al., 2023).

In summary, the diagnosis and management of mitochondrial diseases require a comprehensive approach. Clinicians must be attentive to multisystem involvement and monitor the age-dependent progression of clinical features. Comprehensive multisystem screening, along with dynamic monitoring of neuroimaging findings, lactate levels, and other biomarkers, combined with multi-tissue genetic testing and heteroplasmy analysis, is crucial to avoid misdiagnosis. Through this case and literature review, we aim to enhance the understanding of mitochondrial disorders, emphasizing the importance of early recognition, accurate genetic diagnosis, and timely intervention to improve patient outcomes.

## 5 Conclusion

This report details a diagnostic odyssey of mitochondrial encephalomyopathy associated with the rare m.10158T>C mutation, further complicated by the co-occurrence of a large-scale mtDNA deletion—a genetic combination exceptionally uncommon in MELAS. The case highlights that even in middle-aged patients with vascular risk factors, a presentation of stroke-like episodes warrants consideration of metabolic etiologies beyond common cerebrovascular disease. Definitive diagnosis depended critically on multimodal integration of metabolic profiling (e.g., MRS lactate elevation), tissue-based genetic analysis, and neuroimaging, underscoring the limitations of single-modality or single-tissue testing. Although current management remains symptomatic and cannot reverse established deficits, early recognition and comprehensive evaluation are essential to avoid protracted misdiagnosis, guide appropriate therapeutic intervention, and improve long-term functional outcomes. This case reinforces the necessity of maintaining a high index of suspicion for mitochondrial disorders in adult-onset, stroke-like episodes, regardless of age or conventional risk-factor profiles.

### 5.1 Patient perspective

The patient and her family described that the greatest distress to them was the uncertainty caused by the inability to obtain a definitive diagnosis in the initial stage of the disease. Recurrent seizures made it impossible for the patient to stay alone and rendered her unable to care for herself in daily life. After diagnosing mitochondrial encephalomyopathy and adjusting the treatment regimen based on clinical symptoms, the patient’s seizures were brought under control. Currently, following several months of strict adherence to antiepileptic drug therapy, although some sequelae persist, the patient has regained the ability to communicate normally and live independently, which has rekindled her hope for life.

## Data Availability

The raw data presented in this study are included in the article/supplementary material. For further inquiries, please contact the corresponding author.
